# Intracranial subdural abscess with polymicrobial infections due to frontal sinusitis in an adolescent: life‐threatening complication of a common disease

**DOI:** 10.1002/ccr3.1355

**Published:** 2018-02-04

**Authors:** Arata Hibi, Yoshinobu Amakusa

**Affiliations:** ^1^ Division of Nephrology and Rheumatology Department of Internal Medicine Kariya Toyota General Hospital 5‐15, Sumiyoshi‐cho Kariya Aichi 448‐8505 Japan; ^2^ Department of Neurology Kariya Toyota General Hospital 5‐15, Sumiyoshi‐cho Kariya Aichi 448‐8505 Japan

**Keywords:** Frontal sinusitis, intracranial subdural abscess, polymicrobial infections

## Abstract

Intracranial abscess is one of the most serious complications of frontal sinusitis, particularly among adolescents, even in the absence of odontogenic infection. Polymicrobial infections due to anaerobes are common. Because antibiotic therapy alone is usually ineffective, early endoscopic sinus surgery is the key for infection control and good clinical outcomes.

## Introduction

Sinusitis is one of the most common diseases among primary care patients and usually yields favorable clinical outcomes with or without antibiotic use 1. However, intracranial complications are a life‐threatening manifestation of sinusitis even in the postantibiotic era. Compared with other sinuses, the frontal sinuses are most commonly associated with intracranial infections 2. Polymicrobial infections due to anaerobes are common 3 because of the relatively lower oxygen concentration in the frontal sinus. An early diagnosis and immediate intervention are important for preventing neurological sequelae, particularly in younger patients, given that the average morbidity rate is 27% in pediatric patients (age < 18 years) 3.

## Case Presentation

**Figure 1:**
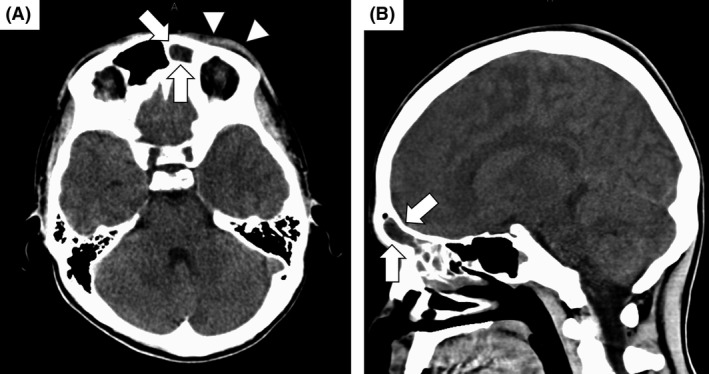
Computed tomography (CT) images of the head on the day of admission. (A and B) Attenuation of the tissue within the left sinus is observed (arrows). Periorbital soft‐tissue thickening is also observed (arrow heads). Bone abnormalities and continuity with the intracranial space are not observed. Intracranial subdural abscess is not apparent on CT images.

**Figure 2:**
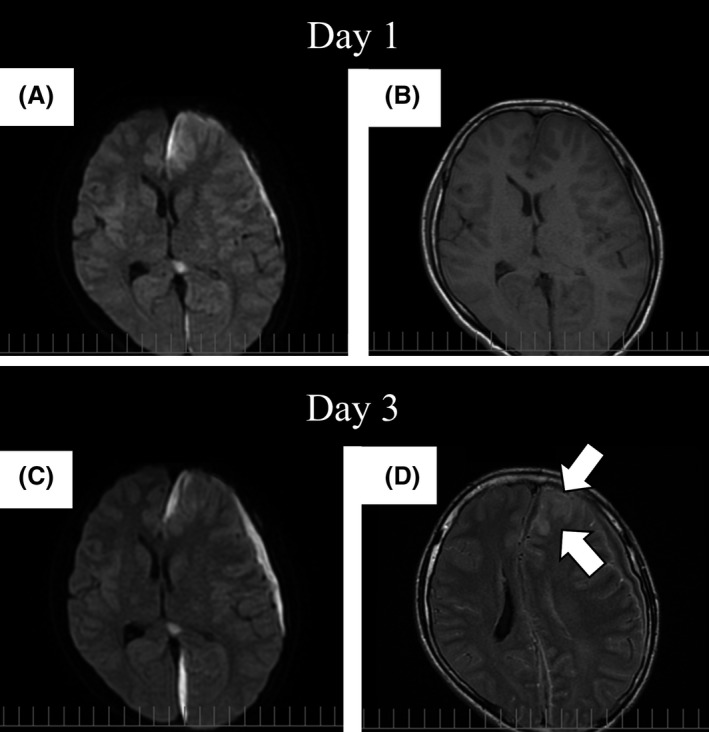
Magnetic resonance imaging studies performed on the day of admission and day 3. (A and C) Despite intravenous antibiotic therapy, enlargement of the subdural abscess is observed in diffusion‐weighted image sequences. (B and D) A high‐intensity area appeared at the left frontal lobe (arrows) in a fluid‐attenuated inversion recovery sequence taken on day 3, and spread of infection was suspected. The midline shift also worsened.

**Figure 3:**
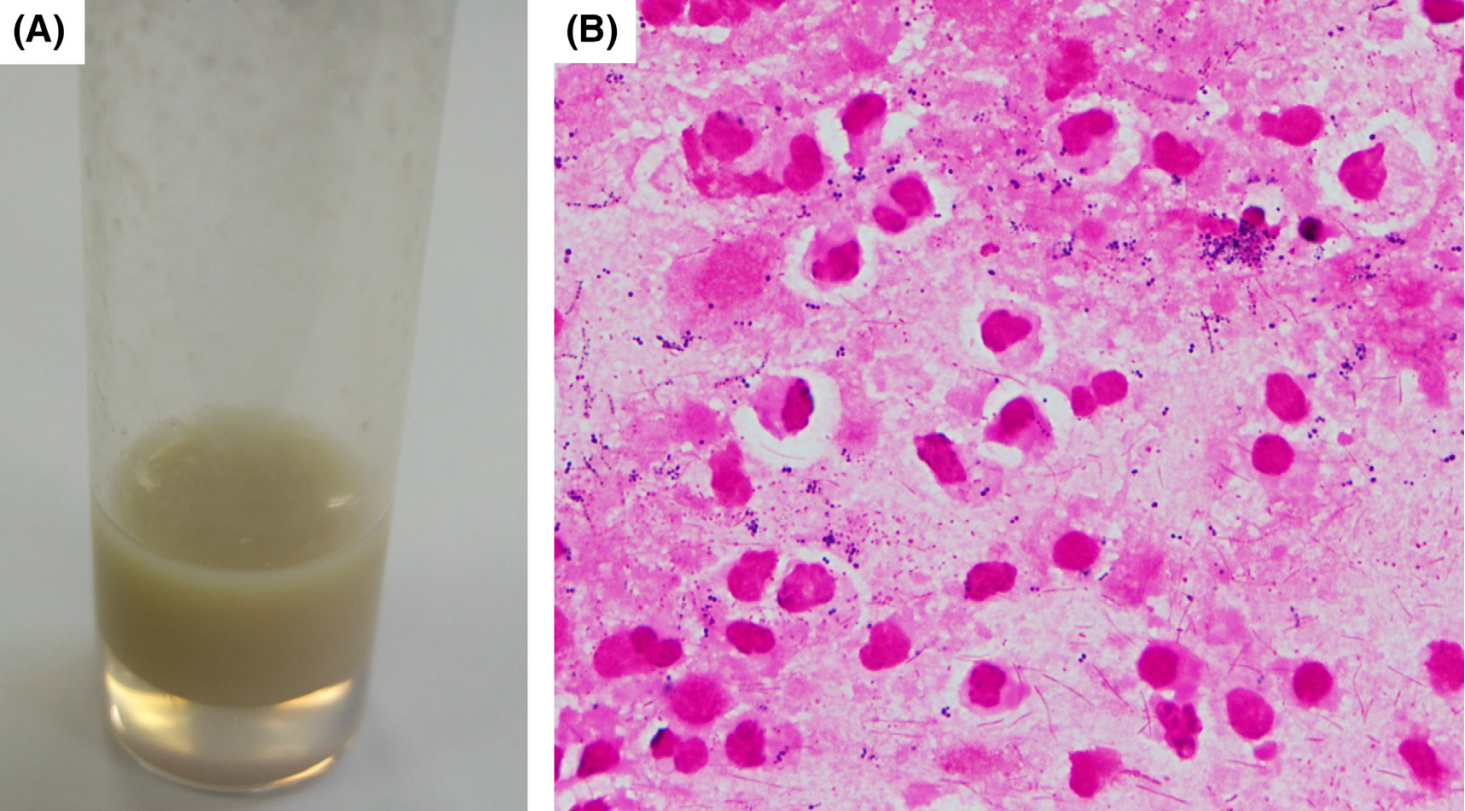
Appearance and Gram staining of the pus obtained during burr hole craniotomy on day 3. (A) Appearance of the pus. (B) Gram staining of the pus (magnification: 1000×) showed various Gram‐positive cocci and Gram‐negative rods; this finding is compatible with polymicrobial infections.

**Figure 4:**
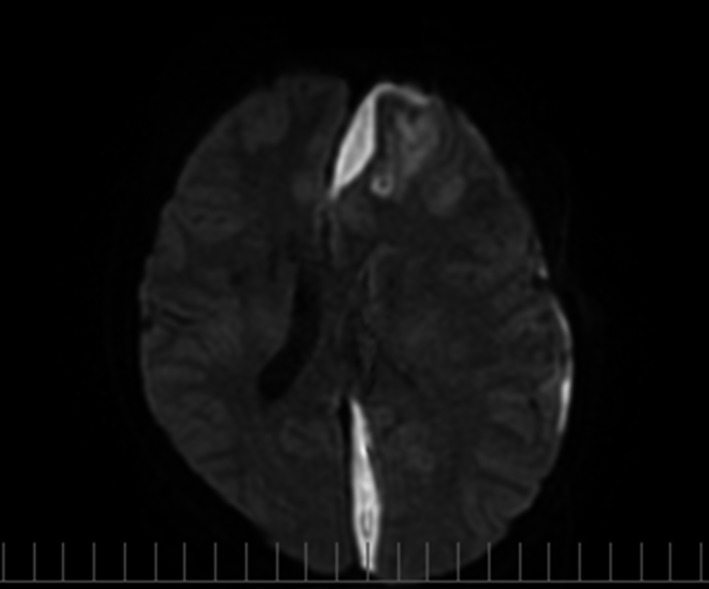
Follow‐up magnetic resonance imaging studies performed on day 7 (4 days after burr hole craniotomy). Diffusion‐weighted image shows enlargement of the subdural abscess without improvement of the midline shift even after burr hole craniotomy.

**Figure 5:**
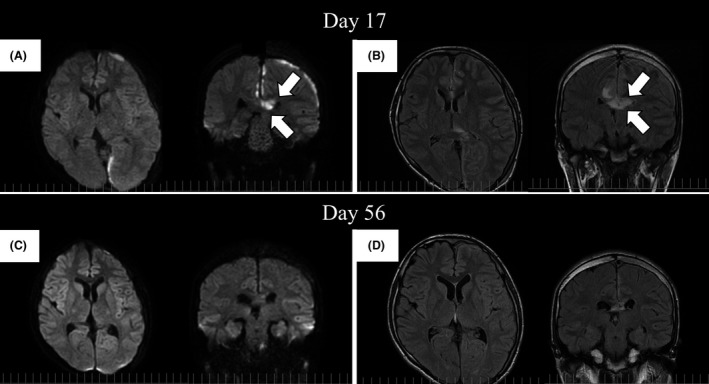
Follow‐up magnetic resonance imaging studies performed on days 17 and 56. (A) The subdural abscess was remarkably diminished in diffusion‐weighted image (DWI) sequences. However, a high‐intensity area appeared in the corpus callosum (arrows) on day 17. (B) In fluid‐attenuated inversion recovery (FLAIR) sequences, the corpus callosum also exhibited high intensity (arrows), and its involvement in the infection was suspected. (C and D) The corpus callosum lesion remarkably improved in both DWI and FLAIR sequences on day 56.

A 16‐year‐old healthy Japanese man visited the emergency department of Kariya Toyota General Hospital, Aichi, Japan, with chief complaints of fever, headache, and nausea. Fever and left‐sided forehead pain occurred 3 days prior to admission, which prompted a visit to the primary care physician. The patient was diagnosed with acute sinusitis, and oral clarithromycin (400 mg/day) was prescribed; however, his symptoms were not relieved. The patient denied any recent dental procedures. At initial presentation, the patient was confused and disoriented. His vital signs were as follows: body temperature, 41.0°C; blood pressure, 129/56 mmHg; heart rate, 108 beats/min; respiratory rate, 30 breaths/min; and oxygen saturation was 96% in room air. His left upper eyelid was slightly swollen. His oral hygiene was good. On neurological examination, the patient exhibited left‐sided palsy. During physical examination, seizure was observed. Meningitis was initially suspected, and lumbar puncture was performed immediately. Cerebrospinal fluid (CSF) analysis showed the following results: appearance, cloudy and yellowish; pressure, >30 mmH_2_O; cell count, 433/μL (polymorphonucleocytes, 88% and monocytes, 12%); protein, 120 mg/dL; and glucose, 62 mg/dL. CSF Gram staining revealed no bacteria, and cultures were negative. Blood test results were as follows: white blood cell count, 10,700/μL and C‐reactive protein levels, 12.14 mg/dL. Blood cultures (1 of 2 sets) were positive for *Fusobacterium nucleatum*. Attenuation of the soft tissue within the left frontal sinus was observed on computed tomography images (Fig. 1). Intracranial subdural abscess was suspected based on magnetic resonance imaging (MRI) findings (Fig. 2). Frontal sinusitis was suspected as the source of infection. Intravenous meropenem (2 g every 8 h) and vancomycin (500 mg every 6 h) were immediately administered as empiric therapy. Because the patient's condition did not improve with antibiotic therapy alone and follow‐up MRI showed enlargement of the abscess (Fig. 2), burr hole craniotomy in the left temporal region was performed on day 3 for abscess drainage. Pus was collected during the surgical procedure, and Gram staining of the pus revealed a polymicrobial pattern (Fig. 3). *Streptococcus constellatus*,* Gemella morbillorum*,* Parvimonas micra*,* Campylobacter showae*, and *Dialister pneumosintes* were isolated from pus cultures. These were identified through matrix‐assisted laser desorption/ionization time‐of‐flight mass spectrometry performed using Microflex LT with a MALDI Biotyper version 3.1 database (Bruker Daltonik, Bremen, Germany), with score values of 2.37, 2.16, 2.26, 1.97, and 2.23, respectively. On day 7, burr hole craniotomy in the left frontal and occipital regions and endoscopic sinus surgery (ESS) were performed to control the infection source because follow‐up MRI showed enlargement of the abscess (Fig. 4). On the same day, the antibiotics were switched to intravenous cefozopran (2 g every 12 h) and metronidazole (500 mg every 6 h). Pus cultures obtained on the same day from the subdural abscess and left frontal sinus were negative. Although corpus callosum involvement was suspected based on findings of the follow‐up MRI performed on day 17, this was resolved, with a size reduction in the subdural abscess and improvement of the midline shift (Fig. 5). The patient was discharged on day 56, and antibiotics were discontinued on the same day. Although slight left‐sided palsy remained, the patient did not experience recurrent symptoms after discharge.

## Discussion

Frontal sinusitis may lead to orbital infections and/or intracranial infections. Intracranial complications include meningitis, subdural abscess, epidural abscess, intracranial abscess, and cavernous thrombophlebitis 3. Subperiosteal abscess of the frontal bone resulting from osteomyelitis secondary to frontal sinusitis and head trauma is known as Pott's puffy tumor (PPT) 4. PPT could be associated with intracranial infections 4. However, intracranial complications may result from frontal sinusitis without osteomyelitis. Communication between the dural venous plexus and the abundant network of the valveless diploic venous system, which contributes to the mucosal venous drainage of the frontal sinus, is thought to be the anatomical reason why frontal sinusitis is frequently associated with intracranial infections 5. Increased blood flow in the diploic veins in accordance with the rapid development of the frontal sinuses in adolescents could explain why teenagers are susceptible to intracranial complications secondary to frontal sinusitis 3, 6. Morino et al. 7 reviewed 70 Japanese patients with sinogenic intracranial infections and reported that it occurs more commonly among males (male: female = 52:18) in the second decade of life. The frontal sinus is the most frequent site of primary infection (83%), and frontal sinusitis is more commonly associated with intracranial abscesses than maxillary, ethmoid, and sphenoid sinusitis. According to their report, sinus surgery is required in 91.4% patients and both sinus surgery and neurosurgery are required in 62.8% patients 7. Garin et al. 8 reported that patients with sinogenic subdural abscess significantly have persistent symptoms and require more surgical procedures than those with epidural abscess; furthermore, ESS has a critical role in surgical management. In the present case, antibiotic therapy alone was ineffective and burr hole craniotomy was initially performed; however, ESS, in addition to second‐time burr hole craniotomy, helped control the infection and stabilized the patient's condition. We believe that ESS played a key role in controlling the infection.

In the present case, multiple organisms were isolated from the blood and pus cultures and some of them have rarely been reported before*. Fusobacterium nucleatum*, an anaerobic Gram‐negative rod, is one of the most abundant species found in the human oral cavity 9. Denes et al. 10 reviewed seven cases of brain abscess caused by *F. nucleatum*, and among them, four (57%) were due to polymicrobial infections. *Streptococcus constellatus*, a facultative Gram‐positive coccus, was reported as one of the causative organisms of intracranial abscess secondary to sinusitis in a previous study 3. *Streptococcus constellatus* is classified as a member of *Streptocossus anginosus* group, which includes *Streptocossus intermedius*,* S. anginosus*, and *S. constellatus*. *Streptococcus constellatus* is found in normal oropharyngeal and gastrointestinal flora. Claridge et al. reported the clinical differences associated with infections caused by these three organisms. They reported that *S. constellatus* and *S intermedius* were more frequently associated with abscess formation compared with *S. anginosus* (76%, 83%, and 19%, respectively); further, *S. constellatus* and *S. anginosus* isolated from abscesses were more frequently associated with polymicrobial infection compared with *S. intermedius* (78%, 100%, and 11%, respectively) 11. Interestingly, Nagashima et al. reported a synergistic effect associated with coinoculation of *S. anginosus* group and *F. nucleatum* with respect to abscess forming ability in an animal experiment model. They reported that the strongest synergetic effect was observed with the combination of *S. constellatus* and *F. nucleatum* 12. *Campylobacter showae* is a motile Gram‐negative straight rod, which prefers an anaerobic atmosphere, rather than a microaerophilic atmosphere, for growth 13. *Campylobacter showae* is usually isolated from the human oral cavity 13 and is more frequently isolated from diseased periodontal sites compared with that from healthy periodontal sites 14. We could not identify previous cases of intracranial abscess caused by *C. showae* in literature. *Gemella morbillorum*, a facultative anaerobic Gram‐positive coccus, is a part of the normal flora of the human oropharynx and gastrointestinal and female genital tracts 15. Although *G. morbillorum* is rarely isolated from human infections, it may cause severe localized and generalized infections such as endocarditis 16, liver abscess 17, pleural empyema 18, and brain abscess 19. Chotai et al. 19 reviewed eight previously reported cases of brain abscess caused by *G. morbillorum* along with their own case; they found that most of the cases (62.5%) were associated with odontogenic infections and previous history of dental procedures. *Parvimonas micra* is an anaerobic Gram‐positive coccus also isolated from patients with brain abscess caused by odontogenic infection 20. *Parvimonas micra* is usually isolated from polymicrobial infections, and dental procedures were found to be risk factors for infection 21. Cobo et al. 21 reviewed previously reported 30 cases of infections caused by *P. micra*; they found spine as the preferred location of infection, whereas intracranial infection was found to be relatively uncommon. *Dialister pneumosintes* is an obligatory anaerobic Gram‐negative rod, which is rarely isolated from human infections. Contreras et al. 22 reported an important role of *D. pneumosintes* in the pathogenesis of human periodontitis. Kogure et al. 23 reported a case of *D. pneumosintes* bacteremia caused by dental caries and sinusitis in a 62‐year‐old Japanese woman who underwent chemotherapy for breast cancer. We could identify only one case of intracranial subdural abscess secondary to sinusitis due to *D. pneumosintes,* which was isolated from blood cultures in literature 24. In that case, similar to our case, the patient was a healthy young man (age, 17 years). In the present case, it should be noted that the patient had intracranial abscess with polymicrobial infections due to frontal sinusitis, with no associated signs of odontogenic infection. The current case also proposes the risk of intracranial complications of sinusitis in healthy young patients and underlines the importance of early surgical management, particularly ESS, if antibiotics therapy alone is not effective.

## Conclusions

Although it is a rare condition, intracranial complications of frontal sinusitis should be considered in clinical settings. We should consider the risk of intracranial complications, particularly in young male patients with frontal sinusitis, even in the absence of odontogenic problems. Early consultation with neurosurgeons and otolaryngologists should be considered because intracranial infections are difficult to treat with antibiotic therapy alone, and ESS could be the key to infection control when the infection source is sinusitis. Early surgical interventions are critical, given the risk of neurological sequelae.

## Conflict of Interest

The authors declare no conflict of interest associated with this manuscript.

## Authorship

AH: responsible for the manuscript. YA: contributed to the critical revision of the manuscript. All authors read and approved the final version of the manuscript.
